# High-efficiency and air-stable P3HT-based polymer solar cells with a new non-fullerene acceptor

**DOI:** 10.1038/ncomms11585

**Published:** 2016-06-09

**Authors:** Sarah Holliday, Raja Shahid Ashraf, Andrew Wadsworth, Derya Baran, Syeda Amber Yousaf, Christian B. Nielsen, Ching-Hong Tan, Stoichko D. Dimitrov, Zhengrong Shang, Nicola Gasparini, Maha Alamoudi, Frédéric Laquai, Christoph J. Brabec, Alberto Salleo, James R. Durrant, Iain McCulloch

**Affiliations:** 1 Department of Chemistry and Centre for Plastic Electronics, Imperial College London, London SW7 2AZ, UK; 2 Department of Physics, Government College University, Lahore 54000, Pakistan; 3 Department of Materials Science and Engineering, Stanford University, 476 Lomita Mall, Stanford, California 94305, USA; 4 Institute of Materials for Electronics and Energy Technology (I-MEET), Friedrich-Alexander-University Erlangen-Nuremberg, 91058 Erlangen, Germany; 5 King Abdullah University of Science and Technology (KAUST), Solar and Photovoltaics Engineering Research Center (SPERC), Thuwal 23955-6900, Saudi Arabia

## Abstract

Solution-processed organic photovoltaics (OPV) offer the attractive prospect of low-cost, light-weight and environmentally benign solar energy production. The highest efficiency OPV at present use low-bandgap donor polymers, many of which suffer from problems with stability and synthetic scalability. They also rely on fullerene-based acceptors, which themselves have issues with cost, stability and limited spectral absorption. Here we present a new non-fullerene acceptor that has been specifically designed to give improved performance alongside the wide bandgap donor poly(3-hexylthiophene), a polymer with significantly better prospects for commercial OPV due to its relative scalability and stability. Thanks to the well-matched optoelectronic and morphological properties of these materials, efficiencies of 6.4% are achieved which is the highest reported for fullerene-free P3HT devices. In addition, dramatically improved air stability is demonstrated relative to other high-efficiency OPV, showing the excellent potential of this new material combination for future technological applications.

## Abstract (web summary)

In organic photovoltaics, the best performing devices usually involve low-bandgap polymers whose limited solubility and stability constrain the scalability of organic solar cells. Here, Holliday *et al*. develop a new acceptor and pair it with canonical P3HT to obtain 6.4% efficient and stable devices.

## Graphical abstract

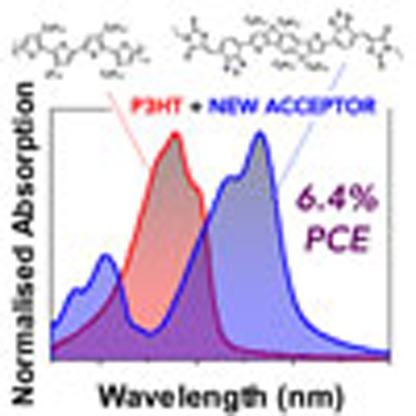

The efficiency of solution-processed organic photovoltaics (OPV) has been increasing rapidly, with the development of new high-performing benzodithiophene^1^,^2^,^3^,^4^ and difluorobenzothiadiazole^5^ -based donor polymers in particular that give up to 10% power conversion efficiency (PCE) combined with fullerene acceptors in single junction cells, and over 11% PCE in tandem devices^6^,^7^. Meanwhile, fullerene-free OPV has also been advancing, driven by the need to find alternative acceptors that overcome the high synthetic costs, limited optical absorption, poor bandgap tunability and morphological instability of fullerene-based acceptors such as phenyl-C_61_-butyric acid methyl ester (PC_60_BM) and its C_71_ analogue PC_70_BM (refs 8, 9, 10). Multiple reports of efficiencies over 6% have now been published with acceptors based on fused ring diimide^11^,^12^,^13^,^14^,^15^ and 1,1-dicyanomethylene-3-indanone^16^,^17^ structures. However, the majority of these record efficiencies are achieved with low-bandgap donor–acceptor polymers such as polythieno[3,4-b]-thiophene-*alt*-benzodithiophene (PTB7), which are known to present intrinsic difficulties to scale-up (thereby increasing costs) as well as suffering from issues with solubility^18^, device irreproducibility and photochemical instability^19^,^20^. Meanwhile, the simple homo-polymer poly(3-hexylthiophene) (P3HT), one of the most extensively used and best understood polymers in OPV research for some time^21^,^22^,^23^, is relatively stable^24^,^25^ and readily scalable due to its straightforward synthesis^26^ and compatibility with high-throughput production techniques^27^. Indeed, P3HT is currently one of the only polymers available in quantities over 10 kg (ref. 23), making it one of the few feasible candidates for commercial OPV, and its use in large-area, roll-to-roll printed solar cells has already been widely demonstrated^28^. Furthermore, the semi-crystalline nature of P3HT, compared with more amorphous polymers, is almost unique in setting an appropriate morphology lengthscale for bulk heterojunction OPV from a range of solvents and processing conditions, as well as providing it with good charge transport properties^29^,^30^,^31^. Despite this, P3HT has been somewhat marginalized in recent years since the introduction of higher efficiency donor–acceptor polymers. For P3HT:PC_60_BM devices, the average efficiency is only around 3% (ref. 21), with a maximum efficiency of 7.4% reported with the more expensive fullerene indene-C_60_-bisadduct (ICBA)^32^. We recently published a new non-fullerene acceptor called (5*Z*,5′*Z*)-5,5′-{(9,9-dioctyl-9*H*-fluorene-2,7-diyl)bis[2,1,3-benzothiadiazole-7,4-diyl(*Z*)methylylidene]}bis(3-ethyl-2-thioxo-1,3-thiazolidin-4-one) (FBR) that had a straightforward and scalable synthesis and gave 4.1% PCE in P3HT devices, which at the time of writing was the highest reported efficiency for a fullerene-free device with P3HT^33^. However, the short-circuit current (*J*_sc_) in these devices was limited by recombination losses arising from the highly intermixed donor and acceptor phases, with FBR apparently unable to aggregate enough to form pure domains that would provide an appropriate charge percolation pathway. In addition, the large extent of spectral overlap of FBR with P3HT and lack of long-wavelength absorption reduced the ability to harvest photons across the spectrum, further limiting the generated photocurrent.

We now present a new acceptor derivative that has been designed to address both the spectral overlap and morphological issues with FBR via replacement of the fluorene core with an indacenodithiophene unit. This has the effect of planarizing the molecular structure and thus significantly red-shifting the absorption as well as increasing the tendency to crystallize on length scales commensurate with charge separation and extraction. We show how these properties can be further tuned via side-chain engineering, with linear (*n*-octyl) alkyl chains yielding a more crystalline material with a further red-shifted absorption onset relative to branched (2-ethylhexyl) chains, resulting in higher *J*_sc_ and PCE. Power conversion efficiencies of up to 6.4% were achieved, which is, to the best of our knowledge, the highest reported for fullerene-free P3HT solar cells. The oxidative stability of these devices is also found to be superior to the benchmark P3HT:PC_60_BM devices, as well as devices with several of the high-performance polymers tested alongside, demonstrating this to be a robust and highly promising new materials combination for OPV.

## Results

### Physical properties

**Figure 1:**
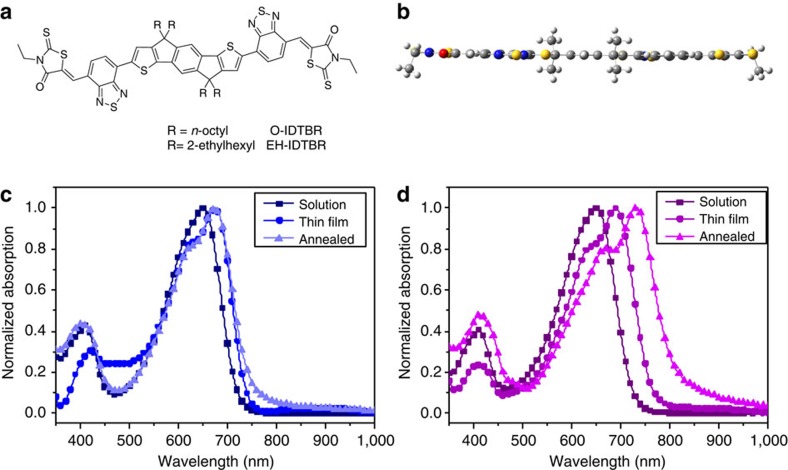
Structure and UV–vis absorption of IDTBR acceptors. (**a**) Chemical structures of O-IDTBR and EH-IDTBR; (**b**) Optimized conformation of IDTBR as calculated by DFT (B3LYP/6–31G*)) with methyl groups replacing alkyl chains for clarity; (**c**,**d**) UV–vis absorption spectra of (**c**) EH-IDTBR and (**d**) O-IDTBR in chloroform solution (1.5 × 10^−5^ mol l^−1^), thin film (spin-coated from 10 mg ml^−1^ chlorobenzene solution) and thin film annealed at 130 °C for 10 min. DFT, density functional theory.

**Figure 2:**
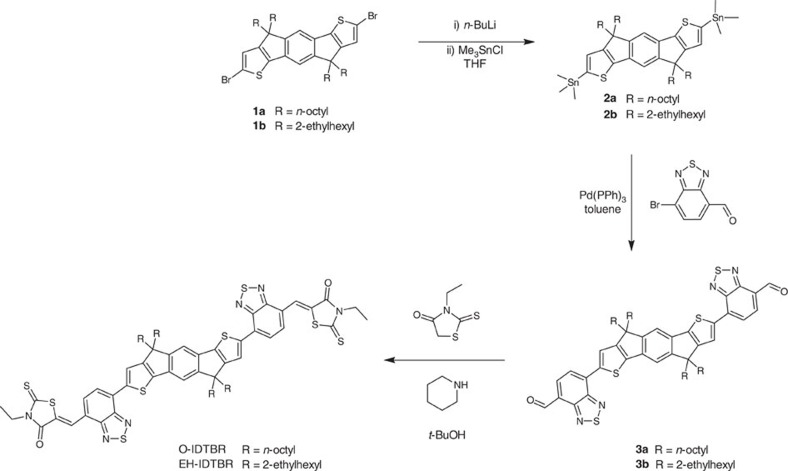
Synthesis of O-IDTBR and EH-IDTBR acceptors. The brominated indacenodithiophene core is first stannylated with trimethyltin chloride, then reacted via Stille coupling with 7-bromo-2,1,3-benzothiadiazole-4-carboxaldehyde. Knoevenagel condensation with 3-ethylrhodanine yields the final product.

The structure of the new IDTBR acceptors is shown in Fig. 1a. The indacenodithiophene (IDT) core was synthesized according to literature procedures^34^,^35^ and alkylated using either linear *n*-octyl (O-IDTBR) or branched 2-ethylhexyl (EH-IDTBR) side chains as shown in Fig. 2. Stille coupling of the stannylated IDT with 7-bromo-2,1,3-benzothiadiazole-4-carboxaldehyde was then followed by Knoevenagel condensation with 3-ethylrhodanine to give O-IDTBR and EH-IDTBR in 60% and 30% final yields, respectively. The acceptors are both stable up to 350 °C (Supplementary Fig. 1) and highly soluble in common organic solvents such as chloroform at room temperature, as well as non-halogenated solvents such as *o*-xylene (60 °C), enabling facile solution processing of OPV devices. In the case of FBR, a torsional angle of 33.7° was calculated between the fluorene core and the adjacent benzothiadiazole unit by density functional theory (DFT) methods. By contrast, IDTBR was calculated to be essentially planar (Fig. 1b) due to the increased quinoidal character of the phenyl-thienyl bond compared with the phenyl–phenyl bond, and the reduced steric twisting from adjacent α-C–H bonds on the coupled phenyl rings^35^,^36^. This enhanced planarity increases conjugation which, when combined with the more electron-rich thiophene-based core, acts to raise the highest occupied molecular orbital (HOMO). This is manifested in a significantly red-shifted UV–visible (UV–vis) absorption spectrum relative to that of FBR. Furthermore, whereas the lowest unoccupied molecular orbital (LUMO) of FBR was localized on the periphery of the molecule, the increased conjugation of IDTBR allows for slightly more delocalization of the LUMO across the central unit (Supplementary Fig. 2), which may be beneficial in terms of molecular oscillator strength and therefore molar absorption coefficient. However, the LUMO of IDTBR is still predominantly located on the periphery of the molecule, which was an important feature in the molecular design as it allows the energy of the highest occupied molecular orbital to be tuned by changing the central unit while preserving the relatively high-lying LUMO energy and thus maintaining a high open-circuit voltage. The molar absorption coefficient of 1 × 10^5^ M^−1^ cm^−1^ (measured in solution) is over twice the value of FBR and demonstrates the potential of these molecules to contribute significantly more to the photocurrent relative to PC_60_BM for which the maximum extinction coefficient in the visible region (400 nm) was measured alongside to be only 3.9 × 10^3^ M^−1^ cm^−1^ in CHCl_3_ (Supplementary Table 1). Furthermore, IDTBR demonstrates significantly stronger absorption in the thin film relative to typical low-bandgap polymers such as PTB7 that absorb at similar wavelengths, as shown from the extinction coefficients plotted in Supplementary Fig. 3a. The absorption coefficient of IDTBR is also higher than those values reported for P3HT^37^,^38^.This introduces an exciting new concept in the design of active layer materials for OPV, where the acceptor can be used as the primary low-bandgap light absorber, able to donate holes on light absorption in at least an equally efficient way as donor polymers traditionally donate electrons on light absorption.

It has been previously shown that the alkyl chain length and degree of branching can have a significant effect on the optoelectronic and aggregation properties in other IDT-BT-based systems^34^ and hence the investigation of both *n*-octyl and 2-ethylhexyl chains with IDTBR. Figure 1c,d compare the UV–vis absorption spectra of the linear O-IDTBR and branched EH-IDTBR. The acceptors have very similar absorption profiles in solution with absorption maxima at 650 nm, and evidently both materials demonstrate greater absorption in the visible region relative to PC_60_BM (Supplementary Fig. 4 and Supplementary Table 1), which further improves their ability to contribute to photocurrent through absorption. In the thin film, the absorption maximum of O-IDTBR is red-shifted by 40 nm relative to that of EH-IDTBR, with a further bathochromic shift of 41 nm for O-IDTBR upon annealing (above 110 °C, see Table 1 and Supplementary Fig. 3b). The shoulder observed at shorter wavelengths, which has been previously attributed to solid-state aggregation in IDT-BT polymers^34^, also becomes more pronounced with thermal annealing. By contrast, the absorption of EH-IDTBR is not affected by annealing (Table 1, Fig. 1c), indicating that the alkyl chains have a significant effect on the tendency of the material to crystallize in the thin film and this in turn strongly affects the absorption properties.

Cyclic voltammetry (CV) in the thin film shows that both EH-IDTBR and O-IDTBR have electron affinity (*EA*) values close to 3.9 eV. The *EA* of P3HT was measured for comparison to be 3.2 eV, allowing sufficient energetic offset for electron transfer between the donor and acceptor. The ionization potential (*IP*) of O-IDTBR was measured to be slightly smaller than that of EH-IDTBR, which accounts for the small difference in optical bandgap (Table 1). This may be due to the enhanced planarization effect of O-IDTBR arising from the additional intermolecular interactions of the more aggregated material. The energy offset between the *IP* of P3HT and both acceptors also appears to be suitable for efficient hole transfer.

### Photovoltaic performance

**Figure 3:**
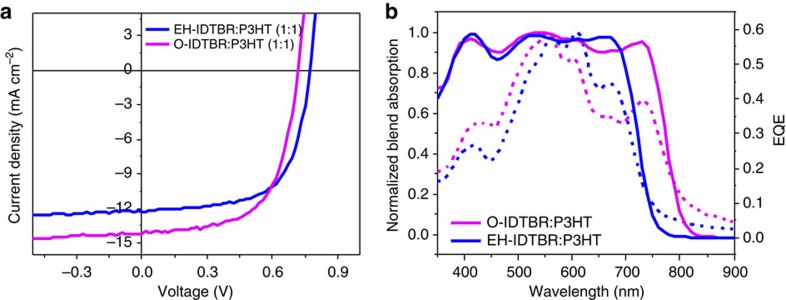
*J–V* characteristics and EQE of IDTBR devices with P3HT. (**a**) *J–V* curves of optimized EH-IDTBR:P3HT and O-IDTBR:P3HT solar cells; (**b**) EQE spectra of optimized EH-IDTBR:P3HT and O-IDTBR:P3HT solar cells (solid lines) alongside normalized thin film absorption spectra of blends (dotted lines).

Solar cells were fabricated using P3HT as the donor polymer due to the favourable energetic offsets mentioned above, as well as its widespread availability of P3HT and its potential for technological scale-up. An inverted device architecture of glass/ITO/ZnO/P3HT:IDTBR/MoO_3_/Ag was chosen for its improved environmental stability relative to the conventional architecture^39^,^40^, allowing for devices to be tested under ambient conditions. The active layer blends (donor-to-acceptor ratio of 1:1) were spin-coated from chlorobenzene solution under ambient conditions without the use of additives. Thermal annealing (10 min at 130 °C) of these films was used to promote ordering of the polymer, as is typical in P3HT solar cells, as well as to induce acceptor crystallization which will be discussed later. Figure 3 and Table 2 show current density–voltage (*J–V*) data for the optimized devices with an active device area of 0.045 cm^2^, which were measured under simulated AM1.5G illumination at 100 mW cm^−2^. Both acceptors yielded high open-circuit voltage (*V*_oc_) values (0.7–0.8 V) relative to reference devices with PC_60_BM as the acceptor, which gave 0.58 V (Supplementary Fig. 5 and Supplementary Table 2) and this difference is accounted for by the smaller electron affinities of the IDTBR acceptors. IDTBR also generates higher short-circuit currents compared to PC_60_BM with P3HT, which may be related to the increased visible wavelength absorption, and therefore greater photocurrent generation, of these new acceptors. A higher average *J*_sc_ of 13.9 mA cm^−2^ is achieved from the O-IDTBR device, compared with 12.1 mA cm^−2^ for EH-IDTBR. This can be understood, at least in part, by the broader external quantum efficiency (EQE) profile of O-IDTBR, which extends beyond 800 nm due to the red-shifted absorption of the acceptor after annealing. Although the *V*_oc_ and fill factor (FF) are both slightly lower for the linear chain analogue, this significantly larger *J*_sc_ leads to an overall increase in average PCE from 6.0% for EH-IDTBR to 6.3% for O-IDTBR, with a maximum PCE of 6.4% for the best performing device. This is among the highest efficiencies for fullerene-free devices as well as being the highest published efficiency for non-fullerene acceptor devices with P3HT. It is also significantly higher than the reference PC_60_BM:P3HT device efficiency of 3.7%, despite the reduced active layer thickness of 75 nm for the IDTBR devices compared with 150 nm for the fullerene-based device. This difference in active layer thickness can also explain the increased peak EQE in the PC_60_BM:P3HT devices as shown in Supplementary Fig. 5b. To explore the compatibility of our new materials with large-area device fabrication, the dependency of *J–V* properties on active area was analysed for O-IDTBR:P3HT devices, as shown in Supplementary Fig. 6 and Supplementary Table 3. For active layers of 0.15 cm^2^, the PCE is maintained at 6.3% and for areas as large as 1.5 cm^2^, the PCE is still relatively high as 4.5%, owing to a slight reduction in *J*_sc_ and FF. It should be noted that these larger area devices were prepared using procedures optimized for the 0.045 cm^2^ cells, and that with further optimization of large-area devices their performance may be further improved, demonstrating these materials to be promising candidates for large-area, scalable OPV.

### Crystal packing

**Figure 4:**
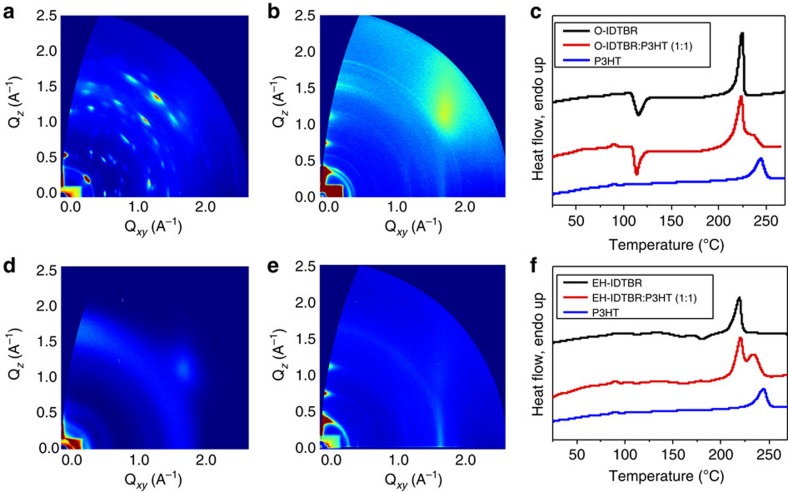
Morphology of acceptors and IDTBR:P3HT blends. (**a**) 2D GIXRD of O-IDTBR; (**b**) 2D GIXRD of O-IDTBR:P3HT (1:1); (**c**) DSC first heating cycles of O-IDTBR, P3HT and 1:1 blend; (**d**) 2D GIXRD of EH-IDTBR thin film; (**e**); 2D GIXRD of EH-IDTBR:P3HT (1:1); and (**f**) DSC first heating cycles of EH-IDTBR, P3HT and 1:1 blend. Thin films for GIXRD were processed using the same conditions as described for optimized devices and DSC drop-cast samples were measured at 5 °C min^−1^. Thermograms are offset vertically for clarity.

As discussed above, one of the limiting factors of the previously published FBR acceptor was the intimately mixed morphology with P3HT due to the amorphous nature of the acceptor, leading to charge recombination losses and limiting device performance. One of the design principles of IDTBR was therefore to increase the planarity of the backbone in order to induce crystallization and the formation of pure acceptor domains. Specular X-ray diffraction (XRD) was used to compare the crystallinity of the acceptors in films that were slightly thicker than those used in device fabrication (280–290 nm) in order to provide enough resolution to observe crystalline reflections by this method. Supplementary Figs 7 and 8 show that, while FBR showed no sign of crystallinity in this case even with annealing, both O-IDTBR and EH-IDTBR give strong diffraction peaks. A clear increase in crystalline order is observed for O-IDTBR after annealing, in accordance with the red-shifted UV–vis absorption. From differential scanning calorimetry (DSC) measurements (Fig. 4) it is apparent that, during the first heating cycle, O-IDTBR undergoes an exothermic crystallization transition with an onset temperature of 108 °C and *T*_c_ of 115 °C. No such thermally induced crystallization occurs during the heating cycle of EH-IDTBR, explaining the different optical response of the acceptors to thermal annealing. DSC measurements were also carried out on drop-cast blends of the acceptors with P3HT to determine the extent of crystallization within the blend. The blend of FBR:P3HT (Supplementary Fig. 8) shows only the melting endotherm for P3HT upon heating, which has been depressed (by 20 °C) and broadened due to the disruption in packing caused by the acceptor; however, no transition for the acceptor is observed which indicates a lack of pure acceptor domains in this blend. By contrast, the heating cycles of O-IDTBR and EH-IDTBR blends with P3HT show the endothermic (and exothermic, in the case of O-IDTBR) transitions from the acceptor as well as the P3HT melt transition, demonstrating that these acceptors are more able to crystallize in the blend than FBR. Furthermore, the melting temperature of P3HT is only depressed by 10 °C in the IDTBR blends, at the same heating rate, suggesting that the crystallization of P3HT is less disrupted by these acceptors.

Grazing incidence XRD (GIXRD) was used to further investigate the formation of pure donor and acceptor domains in the thin-film blends. Figure 4 shows the GIXRD patterns of O-IDTBR and EH-IDTBR in both neat films and in 1:1 blends with P3HT, for which samples were prepared using the same conditions used for solar cells. It is evident that O-IDTBR forms a more ordered film than EH-IDTBR, with a narrow out-of-plane distribution of crystallites as given by the narrow width of the diffraction peaks. In O-IDTBR:P3HT blends, the O-IDTBR crystallites become isotropically distributed and exhibit polycrystalline rings in the diffractogram. The magnitude of the scattering wave vectors of the rings match with the diffraction peaks of neat O-IDTBR as is apparent from the peak analysis shown in Supplementary Figs 9 and 10. This suggests that the presence of P3HT may change the crystallite size and distribution of O-IDTBR but not its lattice structure.

EH-IDTBR has an out-of-plane peak centred at *Q*_z_=1.69 Å^−1^, and several rings in its diffraction pattern. The peak most probably results from a portion of face-on *π–π* stacking of EH-IDTBR aggregates. The rings indicate that besides the aggregates with face-on orientation, the film also has a considerable amorphous fraction. When EH-IDTBR is blended with P3HT, a new peak at *Q*_z_=0.48 Å^−1^ appears, partly overlapping with the broad P3HT (001) alkyl peak at 0.39 Å^−1^. This peak does not correspond to any features seen in the diffraction pattern of neat EH-IDTBR, suggesting that in the presence of P3HT, EH-IDTBR crystallizes in a different orientation or a different polymorph than in neat form, although the diffraction data is not complete enough to allow us to distinguish between these two hypotheses. It should also be noted that the diffraction pattern of P3HT in the blends is the same as that of a pure P3HT film^41^.

### Charge-carrier mobilities

**Figure 5:**
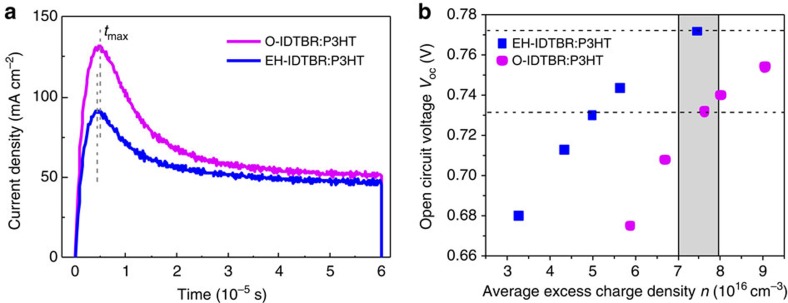
Charge transport and CE of IDTBR:P3HT blends. (**a**) Photo-CELIV of the O-IDTBR:P3HT and the EH-IDTBR:P3HT solar cells at 1 μs delay times; *t*_max_ (the time when the extraction current reaches its maximum value) for O-IDTBR:P3HT and EH-IDTBR:P3HT is 4.7 and 4.3 μs, respectively; (**b**) average charge densities measured in O-IDTBR:P3HT and EH-IDTBR:P3HT devices operating at open circuit as a function of *V*_oc_ determined by CE for different bias light intensities. The grey area marks the data points corresponding ∼1 sun light intensity, and dashed lines correspond to the approximate device *V*_oc_ values (upper line for EH-IDTBR, lower line for O-IDTBR) at 1 sun.

It is well known that charge transport is crucial for efficient OPV devices. Carrier mobility of both donor and acceptor materials can be affected by morphology, field or carrier densities in bulk heterojunction active layers under operating conditions^42^,^43^. To get a reliable charge-carrier mobility of the blend systems, photo-induced charge-carrier extraction in a linearly increasing voltage (photo-CELIV) measurements were conducted. As these photo-CELIV measurements are conducted at 1 sun illumination on actual solar cells, they can provide important information on the transport properties in working devices^44^,^45^. In contrast to single-carrier measurements, the CELIV technique is more sensitive to the faster carrier component in the blend. The average performing EH-IDBTR:P3HT and O-IDTBR:P3HT devices were used in this experiment, having 80–90 nm active layer thickness and 4 mm^2^ active area (see Supplementary Table 4). Figure 5a shows the photo-CELIV transients of the two systems, which were recorded by applying a 2 V per 60 μs linearly increasing reverse bias pulse and a delay time (*t*_d_) of 1 μs. From the measured photocurrent transients, the charge carrier mobility (*μ*) is calculated using the following equation (1):


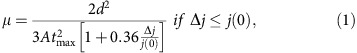


where *d* is the active layer thickness, *A* is the voltage rise speed *A*=d*U*/d*t*, *U* is the applied voltage, *t*_max_ is the time corresponding to the maximum of the extraction peak, and *j*(0) is the displacement current. The photo-CELIV mobilities for the charge carriers in the O-IDTBR and EH-IDTBR blends with P3HT is found to be 5.4±0.4 × 10^−5^ and 5.0±0.3 × 10^−5^ cm^2^ V^−1^ s^−1^ after averaging over various delay times, respectively. The O-IDTBR:P3HT blend shows slightly higher charge-carrier density (which is the integrated area of the photo-CELIV curve at 1 μs delay time) than the branched chain analogue system. In addition to photo-CELIV, the electron mobility of EH-IDTBR:P3HT and O-IDTBR:P3HT blends was determined by space charge-limited current (SCLC) measurements on electron-only devices as well as the hole mobility of EH-IDTBR:P3HT blends on hole-only devices. Both acceptors exhibited electron mobilities ∼3–6 × 10^−6^ cm^2^ V^−1^ s^−1^, while the hole mobility of EH-IDTBR:P3HT was found to be ∼3–7 × 10^−4^ cm^2^ V^−1^ s^−1^ (see Supplementary Fig. 11). Both methods therefore indicate relatively low electron mobilities for these blends. It is interesting to note that in spite of this rather low mobility, IDTBR:P3HT devices display FFs of up to 64% which is within the range of the majority of high-efficiency OPV devices reported in literature^46^. This indicates that non-geminate recombination may be severely suppressed in this system and also that charge generation is not strongly field dependent. However, a more in-depth investigation into the charge recombination dynamics would be needed to determine the exact mechanism behind these high FF values, and these studies are currently on-going.

### Charge extraction

Charge-carrier density (*n*) using charge extraction (CE)^45^,^46^,^47^ measurements were conducted for detailed investigation of the origin of reduced *V*_oc_ in O-IDTBR solar cells compared with branched EH-IDTBR cells with P3HT. All samples were operated at *V*_oc_, but under different background illumination intensities and then shorted in the dark to enable CE. The measured average *n* as a function of *V*_oc_ is depicted in Fig. 5b. It is apparent that, at equivalent charge densities, O-IDTBR devices exhibit ∼40 meV lower open-circuit voltages (see shaded region, corresponding to around 1 sun irradiation) relative to EH-IDTBR. This shift in *n*(*V*_oc_) indicates a 40 meV smaller electronic bandgap for O-IDTBR devices, which is consistent with the reduced open-circuit value (0.73 V) for O-IDTBR:P3HT devices compared with EH-IDTBR:P3HT solar cells (0.77 V). This reduced *V*_oc_ can be explained by the more ordered microstructure of O-IDTBR:P3HT blends, as confirmed with GIXRD measurements, which results in a reduced electronic bandgap in the bulk.

### Photoluminescence (PL) quenching of blends

Photoluminescence (PL) studies were carried out on the EH-IDTBR:P3HT and O-IDTBR:P3HT blends relative to neat reference films of EH-IDTBR, O-IDTBR and P3HT to compare the PL quenching efficiency (PLQE) as shown in Supplementary Fig. 12. The selected range in the PL measurement is mainly focused on the emission of the acceptor. The films were excited at 680 nm to excite selectively the IDTBR acceptors, with the PL quenching being assigned to hole transfer from IDTBR excitons to P3HT. It can be seen that the PL quenching is reasonably efficient for both systems, suggesting efficient hole transfer from acceptor excitons to the P3HT donor polymer. Qualitatively it can be seen that the PLQE is slightly larger for the linear compared with the branched chain system, which further affirms that the increased film crystallinity of O-IDTBR allows for the formation of pure acceptor domains on a lengthscale comparable to the exciton diffusion length of O-IDTBR. We note that this PL quenching contrasts with the almost quantitative acceptor PL quenching that was observed for FBR:P3HT blends, and that this is indicative of more pronounced phase segregation with both IDTBR acceptors compared with FBR^33^.

### Charge generation and recombination dynamics

**Figure 6:**
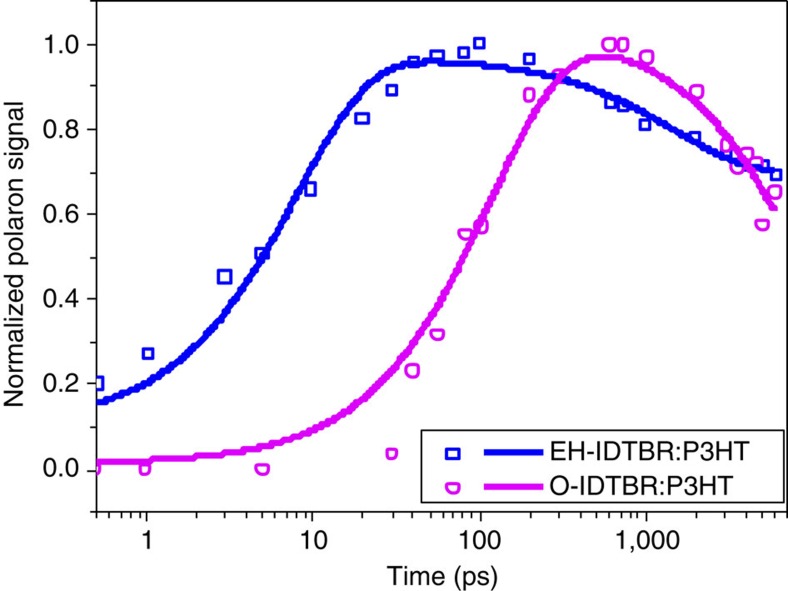
Charge generation and recombination dynamics of IDTBR:P3HT blends. Rise and decay of photogenerated EH-IDTBR and O-IDTBR polaron absorption, obtained by deconvolution of the ultrafast transient absorption spectra of the EH-IDTBR:P3HT and O-IDTBR:P3HT blend films excited at 680 nm, 2 μJ cm^−2^. Symbols correspond to deconvoluted polaron signals and the lines correspond to fitting of the data.

The charge generation process was studied with femtosecond–nanosecond transient absorption spectroscopy (TAS). Transient spectra of EH-IDTBR and O-IDTBR blends, measured with the acceptors excited selectively at 680 nm, are shown in Supplementary Fig. 13. The spectra of neat EH-IDTBR and O-IDTBR films were collected using the same excitation wavelength and density. Because of the spectral overlap of exciton and polaron signals, these spectra were analysed by deconvoluting the blend spectra from the neat P3HT, neat IDTBR and polaron spectra at selected time delays. Successful deconvolution of the blend spectra using the neat data allowed the temporal evolution of the polaron signal to be extracted for both blends studied herein, as shown in Fig. 6. For both blends, polaron growth kinetics were observed on a similar timescale to acceptor exciton decay. This indicates reasonably efficient charge separation from IDTBR excitons and is also consistent with the photocurrent generation from IDTBR light absorption observed in the EQE data (Fig. 3b). The rise of the polaron signal, and decay of acceptor absorption, fitted reasonably well to single exponential functions. For EH-IDTBR:P3HT, the polaron rise kinetics, and decay kinetics of EH-IDTBR exciton absorption, primarily exhibit time constants of 10–20 ps. Only a small fraction (10–20%) of the polaron generation appears to occur within our instrument response. This contrasts with FBR:P3HT blends, where at least 50% of polaron generation was observed to be instrument response limited^33^, consistent with more complete phase segregation compared with FBR. Slower polaron formation and exciton decay is observed for O-IDTBR:P3HT (60–120 ps), indicating more delayed polaron generation for this blend which is consistent with our PLQE results. We have previously reported relatively slow (hundreds of picoseconds) polaron generation from acceptor excitons in polymer:PCBM blends, and correlated these with exciton diffusion within pure PCBM domains to the donor/acceptor interface^47^. It appears likely that the slow polaron generation kinetics we observe herein are also limited by the kinetics of exciton diffusion within pure IDTBR domains, with the slower kinetics observed for O-IDTBR being consistent with increased phase separation for this blend as discussed above. Charge recombination is also apparent in Fig. 6 as a decay of the polaron signal at longer time delays. It is apparent that these kinetics are slower for O-IDTBR compared with EH-IDTBR, again most probably associated with great phase segregation in the O-IDTBR blend.

### Solar cell stability

**Figure 7:**
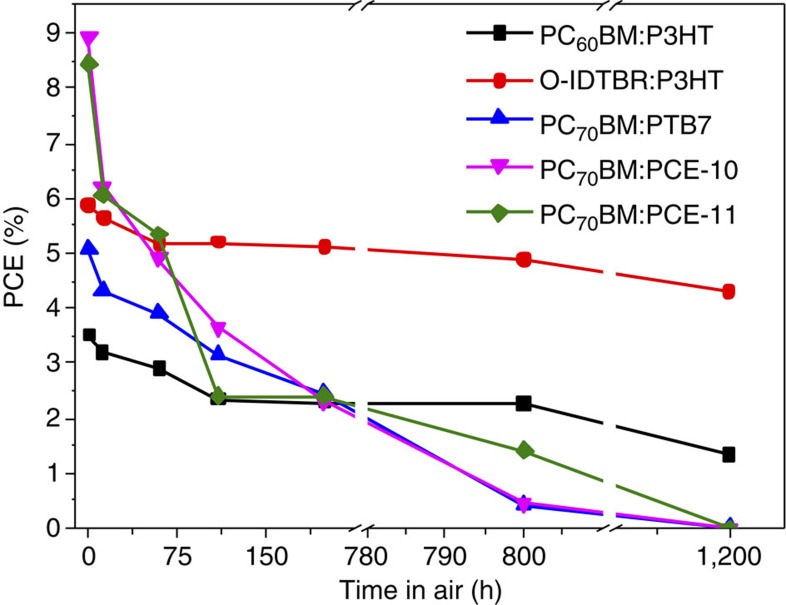
Solar cell stability. Oxidative stability of O-IDTBR:P3HT device efficiencies (PCE) compared with other high-performance polymer:fullerene systems. Devices were stored in the dark under ambient conditions between measurements.

Oxidative stability is an essential consideration for the technological implentation of OPV materials^24^. For many of the record high efficiencies reported with low-bandgap polymers, all device fabrication and measurement must be carried out in inert conditions to maintain this performance. By contrast, the efficiencies reported herein for IDTBR:P3HT were obtained with device processing and measurement carried out in air (except for active layer annealing in a nitrogen glovebox). This improved stability is partially attributed to the inverted architecture used, which means that no encapsulation steps are needed for these devices. To further investigate the stability of IDTBR:P3HT devices to air, aging measurements were carried out alongside reference devices of PC_60_BM:P3HT as well as three of the most widely reported high-efficiency polymers PTB7, PCE-10 (PTB7-Th) and PCE-11 (PffBT4T-2OD)^5^,^48^,^49^ For a fair comparison, all devices were prepared in the same inverted architecture as for IDTBR devices. After the initial (stabilized PCE) measurement was taken, devices were stored in the dark under ambient conditions between measurements, which were taken at intervals over the course of 1,200 h. The corresponding PCE data is shown in Fig. 7, with normalized data given in Supplementary Fig. 14 along with the polymer structures. It is clear from this data that O-IDTBR:P3HT devices demonstrate the least degradation out of the materials studied, and that after an initial small drop in performance within the first 60 h, the PCE remains relatively stable and still gives 73% of the initial PCE even after 1,200 h. By contrast, the efficiency of the high-performance donor polymer devices deteriorates remarkably quickly and has fallen to zero by the end of the period of study. This further demonstrates the potential of our new acceptor design for stable, scalable solar cells with practical operating lifetimes, and also gives strong support for the choice of P3HT as donor polymer in these devices.

In addition to oxidative stability, the morphological stability of the O-IDTBR:P3HT blends was investigated. One of the main issues with fullerene-based acceptors like PC_60_BM is that large-scale aggregates and crystals emerge from the meta-stable blend morphology over time. This process can be monitored by polarized optical microscopy during accelerated aging of the films upon annealing^29^,^50^. To compare the thermal aging of the IDTBR blends with fullerene blends, films of O-IDTBR:P3HT and PC_60_BM:P3HT were prepared on ZnO/ITO substrates and these were subjected to annealing at 140 °C for 1 h. As the micrographs in Supplementary Fig. 15 show, large (1–20 μm) aggregates appear after 1 h annealing of the fullerene blend, whereas the O-IDTBR blend remains smooth and featureless after annealing, suggesting that this new acceptor offers improved morphological stability over fullerene acceptors, at least in terms of lateral diffusion.

## Discussion

In this work, we present a new small molecule electron acceptor IDTBR that is based on an indacenodithiophene core with benzothiadiazole and rhodanine flanking groups. IDTBR is designed to give high performance with the donor polymer P3HT, chosen for its commercial scale-up potential both in terms of cost, scalability and stability. In comparison with our previously published acceptor FBR, which had an essentially overlapping absorption profile with P3HT, this new acceptor has a significantly reduced optical bandgap owing to the more planar molecular backbone, delocalized electronic structure and push–pull molecular orbital hybridization, resulting in a UV–vis absorption profile that is now highly complementary to that of P3HT. This gives broader photon harvesting across the incident solar spectrum within the active layer, which is reflected in higher short-circuit currents and power conversion efficiencies relative to FBR:P3HT devices. Furthermore, the absorption onset of this new IDTBR acceptor can be tuned by judicial choice of solubilizing alkyl chains on the IDT unit. Linear (O-IDTBR) chains promote stronger intermolecular packing, which is particularly enhanced by thermal annealing, relative to branched (EH-IDTBR) chains. One effect of this is to further red-shift the absorption of O-IDTBR relative to the branched counterpart, which results in a broader EQE profile, higher *J*_sc_ and an increase in PCE from 6.0 to 6.4%. CE measurements at the same light intensity reveal a reduced electronic bandgap for O-IDTBR relative to EH-IDTBR, which explains the difference in *V*_oc_ measured for these devices. As well as affecting the optoelectronic properties, the enhanced intermolecular interactions of the linear alkyl chain also have an effect on the blend morphology. Relative to FBR, both IDTBR acceptors exhibit increased crystallinity and, crucially, formation of pure acceptor domains as evidenced by GIXRD and DSC studies. O-IDTBR in particular shows pronounced crystal packing upon annealing, which is consistent with the reduced optical bandgap. This results in greater phase segregation for the linear analogue which is manifested in reduced PL quenching of the acceptor emission, as well as a delayed polaron generation and slower recombination dynamics in the O-IDTBR:P3HT blend. Interestingly, the charge-carrier mobilities measured for the IDTBR:P3HT blends appear quite low, considering the reasonably high FFs obtained from devices (up to 64%) and the charge recombination dynamics of these systems therefore warrant further investigation to determine whether non-geminate recombination is significantly suppressed. In addition to high efficiencies, IDTBR:P3HT devices demonstrate improved stability in ambient conditions compared with the benchmark PC_60_BM:P3HT device, as well as several systems with typical low-bandgap, high-performance polymers, which were found to degrade at a dramatic rate when exposed to air. IDTBR devices also showed improved morphological stability to fullerene devices in accelerated aging studies. These results strongly supports the use of P3HT, in conjunction with non-fullerene acceptors such as IDTBR, for high-efficiency, scalable and stable OPV for future technological applications.

## Methods

### General characterization

^1^H and ^13^C NMR spectra were collected on a Bruker AV-400 spectrometer at 298 K and are reported in p.p.m. UV–vis absorption spectra were recorded on a UV-1601 Shimadzu UV–vis spectrometer. DSC experiments were carried out with a Mettler Toledo DSC822 instrument at a heating rate of 5 °C min^−1^ under nitrogen. Samples were prepared by drop-casting the materials from CHCl_3_ solution directly into the DSC pan and allowing the solvent to evaporate under Ar. Specular XRD was carried out on thin films of the acceptors spin-coated from CHCl_3_ solutions (30 mg ml^−1^, 600 r.p.m.) using a PANalytical X'Pert PRO MRD diffractometer equipped with a nickel-filtered Cu-Kα1 beam and X'Celerator detector, with a current *I*=40 mA and accelerating voltage *U*=40 kV. Samples for GIXRD were spin-coated on Si (100) substrates following the same spin-coating and annealing procedures as were used in fabricating solar cells.

### Synthesis

The compounds **1a** and **1b** were prepared according to literature procedure^34^,^35^, as was 7-bromo-2,1,3-benzothiadiazole-4-carboxaldehyde^33^. P3HT was obtained from Flexink Ltd. All other reagents and solvents were purchased from Sigma Aldrich or Acros Organics and used as received. All reactions were carried out using conventional Schlenk techniques in an inert argon atmosphere.

*2a.* A solution of **1a** (2.11 g, 2.42 mmol) in anhydrous tetrahydrofuran (200 ml) was stirred at −78 °C for 30 min. *n*-BuLi (2.42 ml, 6.04 mmol, 2.5 M in hexanes) was added dropwise and the solution was stirred at −78 °C for 30 min followed by −10 °C for 30 min. After cooling again to −78 °C, trimethyltin chloride was added (7. 26 ml, 7.56 mmol, 1 M in hexanes) and the solution was allowed to return to room temperature overnight. The reaction was then poured into water and extracted with hexane, washed successively with acetonitrile to remove excess trimethyltin chloride and dried over MgSO_4_ to yield **2a** as a yellow oil (2.18 g, 86%). ^1^H NMR (400 MHz, CDCl_3_) *δ*: 7.25 (s, 2H), 6.97 (s, 2H), 1.97–1.91 (m, 4H), 1.86–1.78 (m, 4H), 1.23–1.05 (m, 48H), 0.83–0.80 (t, 12H, *J*=7 Hz), 0.39 (s, 18H); ^13^C NMR (101 MHz, CDCl_3_) *δ*: 157.15, 153.47, 147.71, 139.24, 135.31, 129.55, 113.42, 53.06, 39.20, 31.87, 30.07, 30.03, 29.31, 24.17, 22.68, 14.14 and −8.02. MS (ES-ToF): *m/z* calculated for C_54_H_90_S_2_Sn: 1,040.45; *m/z* found 1,041.40 (M+H)^+^.

*3a.* A solution of **2a** (1.04 g, 1.0 mmol) and 2,1,3-benzothiadiazole-4-carboxaldehyde (0.73 g, 3.0 mmol) in anhydrous toluene (40 ml) was degassed for 45 min before Pd(PPh_3_)_4_ (58 mg, 0.05 mmol) was added and this solution was heated at 100 °C overnight. The reaction mixture was then cooled and purified by flash column chromatography on silica mixed with potassium fluoride using CHCl_3_ as the eluent. Further purification by column chromatography on silica using CH_2_Cl_2_/pentane (1:1) followed by precipitation from methanol yielded **3a** as a dark purple solid (0.93 g, 90%). ^1^H NMR (400 MHz, CDCl_3_) *δ*: 10.72 (s, 2H), 8.27 (s, 2H), 8.25 (d, *J*=7.7 Hz, 2H), 8.06 (d, *J*=7.5 Hz, 2H), 7.45 (s, 2H), 2.05 (dtd, *J*=59.3, 12.9, 4.6 Hz, 8H), 1.05–1.2 (m, 38H), 0.99–0.81 (m, 10H), 0.77 (t, *J*=6.8 Hz, 12H); ^13^C NMR (101 MHz, CDCl_3_) *δ*: 188.44, 157.04, 154.02, 152.29, 147.00, 140.67, 136.44, 134.14, 132.87, 131.62, 124.87, 124.8, 122.80, 114.12, 54.43, 39.16, 31.79, 29.98, 29.29, 29.20, 24.29, 22.58, 14.04. MS (ES-ToF): *m/z* calculated for C_62_H_78_N_4_O_2_S_4_: 1,038.5; *m/z* found 1,041.40.

*O-IDTBR.* **3a** (0.40 g, 0.39 mmol) and 3-ethylrhodanine (186 mg, 1.16 mmol) were dissolved in *tert*-butyl alcohol (30 ml). Two drops of piperidine were added and the solution was left to stir at 85 °C overnight. The product was extracted with CHCl_3_ and dried over MgSO_4_. The crude product was purified by flash column chromatography on silica in CH_2_Cl_2_ and precipitated from methanol. The precipitate was collected and dried by vacuum filtration to afford **O-IDTBR** a dark blue solid (0.40 g, 78%). mp=219–221 °C. ^1^H NMR (400 MHz, CDCl_3_) *δ*: 8.54 (s, 2H), 8.24 (s, 2H), 8.03 (d, *J*=8.0 Hz, 2H), 7.74 (d, *J*=7.9 Hz, 2H), 7.45 (s, 2H), 4.27 (q, *J*=8.0 Hz, 4H), 2.18–1.96 (m, 8H), 1.35 (t, *J*=8.1 Hz, 6H), 1.22–1.12 (m, 40H), 0.99–0.90 (m, 8H), 0.80 (m, 12H). ^13^C NMR (101 MHz, CDCl_3_) *δ*: 193.04, 167.59, 157.05, 154.63, 154.22, 151.77, 146.15, 141.02, 136.41, 131.37, 130.54, 127.29, 124.49, 124.25, 124.08, 123.82, 113.97, 54.38, 39.94, 39.19, 31.82, 30.02, 29.33, 29.24, 24.30, 22.61, 14.08 and 12.35. MS (matrix-assisted laser desorption/ionization–time of flight): *m/z* calculated for C_72_H_88_N_6_O_2_S_8_: 1,324.5; m/z found 1,326.0 (M+H)^+^.

*2b.* A solution of **1b** (1.09 g, 1.25 mmol) in anhydrous tetrahydrofuran (40 ml) was stirred at −78 °C for 30 min. *n*-BuLi (1.25 ml, 3.12 mmol, 2.5 M in hexanes) was added dropwise and the solution was stirred at −78 °C for 1 h. Trimethyltin chloride was then added (3.75 ml, 3.75 mmol, 1 M in hexanes) and the solution was allowed to return to room temperature overnight. The reaction was then poured into water and extracted with hexane, washed successively with acetonitrile to remove excess trimethyltin chloride and dried over MgSO_4_ to yield **2b** as a yellow oil (1.16 g, 89%). ^1^H NMR (400 MHz, CDCl_3_) *δ*: 7.28 (s, 2H), 6.99 (s, 2H), 1.96–1.88 (m, 8H), 1.87–1.82 (m, 8H), 0.99–0.46 (m, 60H), 0.37 (s, 18H). ^13^C NMR (101 MHz, CDCl_3_) *δ*: 157.40, 153.43, 147.51, 140.73, 135.20, 130.04, 113.95, 53.52, 43.59, 34.89, 32.20, 29.75, 28.74, 28.10, 22.67, 14.16 and −8.16.

*3b.* A solution of **2b** (0.94 g, 0.90 mmol) and 2,1,3-benzothiadiazole-4-carboxaldehyde (0.53 g, 2.17 mmol) in anhydrous toluene (30 ml) was degassed for 45 min before Pd(PPh_3_)_4_ (52 mg, 0.05 mmol) was added and this solution was heated at 110 °C overnight. The reaction mixture was then cooled and purified by flash column chromatography on silica mixed with potassium fluoride using CHCl_3_ as the eluent. Further purification by column chromatography on silica using CH_2_Cl_2_/pentane (1:1) followed by precipitation from methanol yielded **3b** as a dark purple solid (0.40 g, 43%). ^1^H NMR (400 MHz, CDCl_3_) *δ*: 10.72 (s, 2H), 8.37–8.30 (m, 2H), 8.25 (d, *J*=7.6 Hz, 2H), 8.03 (d, *J*=7.5 Hz, 2H), 7.49 (s, 2H), 2.15–2.05 (m, 8H), 1.05–0.85 (m, 40H), 0.74–0.50 (m, 20H). MS (ES-ToF): *m/z* calculated for C_62_H_78_N_4_O_2_S_4_: 1,038.50; *m/z* found 1,038.50 (M^+^).

*EH-IDTBR.* **3b** (0.20 g, 0.19 mmol) and 3-ethylrhodanine (93 mg, 0.58 mmol) were dissolved in *tert*-butyl alcohol (15 ml). 1 drop of piperidine was added and the solution was left to stir at 85 °C overnight. The product was extracted with CHCl_3_ and dried over MgSO_4_. The crude product was purified by flash column chromatography on silica with CH_2_Cl_2_ as the eluent followed by precipitation from methanol to yield **EH-IDTBR** as a dark blue solid (0.20 g, 80%). mp=218–220 °C. ^1^H NMR (400 MHz, CDCl_3_) *δ*: 8.53 (s, 2H), 8.27 (m, 2H), 7.99 (m, 2H), 7.73 (d, *J*=8.1 Hz, 2H), 7.47 (s, 2H), 4.25 (q, *J*=8.0 Hz, 4H), 2.07 (m, 8H), 1.34 (t, *J*=8.0 Hz, 6H), 0.95–0.90 (m, 36H), 0.69–0.54 (m, 24H). ^13^C NMR (101 MHz, CDCl_3_) *δ*: 193.07, 167.58, 156.76, 154.63, 153.93, 151.80, 146.14, 140.46, 136.38, 131.37, 130.64, 127.31, 125.08, 124.51, 124.30, 123.73, 114.82, 54.19, 39.94, 35.13, 34.16, 28.64, 28.25, 27.26, 22.86, 14.18, 12.33 and 10.60. MS (matrix-assisted laser desorption/ionization–time of flight): *m/z* calculated for C_72_H_88_N_6_O_2_S_8_: 1,324.5; *m/z* found 1,325.9 (M+H)^+^.

### Cyclic voltammetry

CV measurements were performed using an Autolab PGSTAT101 potentiostat. Thin films of the acceptor were spin-coated onto ITO-coated glass substrates to be used as the working electrode, alongside a platinum mesh counter electrode and Ag/Ag^+^ reference electrode. Measurements were carried out in anhydrous and deoxygenated acetonitrile with 0.1 M of tetrabutylammonium hexafluorophosphate (TBA PF_6_) as the supporting electrolyte, and calibrated against ferrocene in solution using a cylindrical Pt working electrode. IP and EA values were calculated from the following equations:


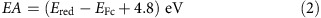



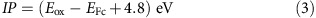


where *E*_red_ and *E*_ox_ are taken from the onset of reduction and oxidation, respectively, and *E*_Fc_ is taken as the half-wave potential of ferrocene.

### OPV devices

Bulk heterojunction solar cells were fabricated with an inverted architecture (glass/ITO/ZnO/P3HT:Acceptor/MoO_3_/Ag). Glass substrates were used with pre-patterned indium tin oxide (ITO). These were cleaned by sonication in detergent, deionized water, acetone and isopropanol, followed by oxygen plasma treatment. ZnO layers were deposited by spin-coating a zinc acetate dihydrate precursor solution (60 μl monoethanolamine in 2 ml 2-methoxyethanol) followed by annealing at 150 °C for 10–15 min, giving layers of 30 nm. The P3HT:IDTBR (1:1 ratio by mass) active layers were deposited from 24 mg ml^−1^ solutions in chlorobenzene by spin-coating at 2,000 r.p.m., followed by annealing at 130 °C for 10 min. Active layer thicknesses were 75 nm (averaged over six devices) for both acceptor blends. P3HT:PC_60_BM (1:1 ratio by mass) layers were spin-coated at 1,500 r.p.m. from 40 mg ml^−1^ solutions in o-dichlorobenzene, followed by annealing in the glovebox at 130 °C for 20 min, resulting in active layer thicknesses of 148 nm. MoO_3_ (10 nm) and Ag (100 nm) layers were deposited by evaporation through a shadow mask yielding active areas of 0.045 cm^2^ in each device. (*J–V*) characteristics were measured using a Xenon lamp at AM1.5 solar illumination (Oriel Instruments) calibrated to a silicon reference cell with a Keithley 2400 source meter, correcting for spectral mismatch. Incident photon conversion efficiency was measured by a 100 W tungsten halogen lamp (Bentham IL1 with Bentham 605 stabilized current power supply) coupled to a monochromator with computer controlled stepper motor. The photon flux of light incident on the samples was calibrated using a UV-enhanced silicon photodiode. A 590-nm long-pass glass filter was inserted into the beam at illumination wavelengths longer than 580 nm to remove light from second-order diffraction. Measurement duration for a given wavelength was sufficient to ensure the current had stabilized.

The low-bandgap polymers PTB7, PCE-10 (PTB7-Th) and PCE-11 (PffBT4T-2OD) used in stability studies were obtained from Ossila, and the active layers for these devices were prepared as follows, with the same architecture a used for the IDTBR:P3HT devices.

### PTB7:PC_70_BM

Active layer solutions (D:A ratio 1:1.5) were prepared in CB with 3 wt% 1,8-diiodooctane (total concentration 25 mg ml^−1^). To completely dissolve the polymer, the active layer solution was stirred on a hot plate at 80 °C for at least 3 h. Active layers were spin-coated from the warm polymer solution on preheated substrates in a nitrogen glove box at 1,500 r.p.m.

### PCE-10:PC_70_BM

Active layer solutions (D:A ratio 1:1.5) were prepared in CB with 3 wt% 8-diiodooctane (total concentration 35 mg ml^−1^). To completely dissolve the polymer, the active layer solution was stirred on a hot plate at 80 °C for at least 3 h. Active layers were spin-coated from the warm polymer solution onto preheated substrates in a nitrogen glove box at 1,500 r.p.m.

### PCE-11:PC_70_BM

Active layer solutions (D:A ratio 1:1.4) were prepared in CB/o-DCB (1:1 volume ratio) with 3 wt% 8-diiodooctane (polymer concentration: 10 mg ml^−1^). To completely dissolve the polymer, the active layer solution was stirred on a hot plate at 110 °C for at least 3 h. Active layers were spin-coated from the warm polymer solution onto preheated substrates in a nitrogen glove box at 1,000 r.p.m.

### Photo-CELIV

In photo-CELIV measurements, the devices were illuminated with a 405 nm laser-diode. Current transients were recorded across an internal 50 Ω resistor on an oscilloscope (Agilent Technologies DSO-X 2024A). A fast electrical switch was used to isolate the cell and prevent CE or sweep out during the laser pulse and the delay time. After a variable delay time, a linear extraction ramp was applied via a function generator. The ramp, which was 20 μs long and 2 V in amplitude, was set to start with an offset matching the *V*_oc_ of the cell for each delay time. The geometrical capacitance is calculated as:


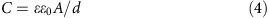


where *A* is the device area (4 mm^2^), *ɛ*=3 and, *ɛ*_0_=8.85 × 10^−12^ F m^−1^ are the relative and absolute dielectric permittivity, respectively, and *d* is the active layer thickness (90 nm). *C* is then calculated as 1 nF. Assuming *R*_load_=50 nm, the *RC* value is 5.9 × 10^−8^ s. Assuming the electrical field (*E*) is 1 × 10^5^ V m^−1^, the transient time (*t*) is calculated with the following formula:


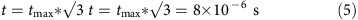


### Charge extraction

In CE measurements, the devices were illuminated in air with a 405 nm laser diode for 200 μs, which was sufficient to reach a constant open-circuit voltage with steady state conditions. At the end of the illumination period, an analogue switch was triggered that switched the solar cell from open-circuit to short-circuit (50 ω) conditions within less than 50 ns. By adjusting the laser intensity, different open-circuit voltages were obtained which allowed a plot to be generated of charge-carrier density over voltage. As described by Shuttle *et al*.^51^, a correction was applied for the charge on the electrodes that results from the geometric capacity of the device^52^.

### Space charge-limited current

SCLC measurements were performed on electron-only devices of the structure ITO/PEDOT:PSS/Al/P3HT:acceptor/Al and on hole-only devices of the structure ITO/PEDOT:PSS/P3HT:acceptor/Au using a Paios (FLUXiM AG) measurement system. The current–voltage characteristics were fitted by the Mott–Gurney law in the region where the current follows the square of the voltage to extract the carrier mobility.

### PL spectroscopy and transient absorption spectroscopy (TAS)

Samples for TAS and PL spectroscopy were spin-coated onto glass using the same conditions as for solar cells. Spectra were measured using a steady state spectrofluorimeter (Horiba Jobin Yvon, Spex Fluoromax 1). The spin-coated films were excited at 680 nm. Sub-picosecond TAS was carried out at 800 nm laser pulse (1 kHz, 90 fs) by using a Solstice (Newport Corporation) Ti:sapphire regenerative amplifier. A part of the laser pulse was used to generate the pump laser at 680 nm, 2 μJ cm^−2^ with a TOPAS-Prime (light conversion) optical parametric amplifier. The other laser output was used to generate the probe light in near visible continuum (450–800 nm) by a sapphire crystal. The spectra and decays were obtained by a HELIOS transient absorption spectrometer (450–1,450 nm) and decays to 6 ns. The samples were measured in N_2_ atmosphere. Deconvolution of the blend spectra was conducted by fitting the singlet EH-IDTBR exciton spectrum (S_exciton_) and the P3HT:EH-IDTBR polaron spectrum (S_polaron_) at 6 ns to the blend spectra for 20 different time delays using the equation:


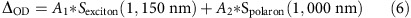


where *A*_1_ and *A*_2_ are linear coefficients that estimate the percentage contribution of the spectra to the experimental blend spectra. *S*_exciton_ was derived from the transient absorption spectra of the EH-IDTBR, which peaks at 1,150 nm at selected time delays (Supplementary Fig. 13). This signal, assigned to singlet exciton absorption, disappeared at ∼20 ps (Fig. 6). The polaron spectrum was derived from the TA spectra of the blend at 6 ns, where no exciton contributions are expected (Supplementary Fig. 13). Note that an exciton signal from P3HT is not expected, as supported by our PL measurements.

## Additional information

**How to cite this article:** Holliday, S. *et al*. High-efficiency and air-stable P3HT-based polymer solar cells with a new non-fullerene acceptor. *Nat. Commun.* 7:11585 doi: 10.1038/ncomms11585 (2016).

## Supplementary Material

Supplementary Information Supplementary Figures 1-15 and Supplementary Tables 1-4

## Figures and Tables

**Table 1:** Optoelectronic properties of O-IDTBR and EH-IDTBR acceptors.

	***ɛ*** **(10**^4^** M**^−1^** cm**^−1^)*	***λ***_**max**_ solution (nm)*	***λ***_**max**_ **film (nm)**†	***λ***_**max**_ **ann. (nm)**‡	***E***_**g**_ **opt. (eV)**†	***EA*** **(eV)**§	***IP*** **(eV)**||
O-IDTBR	9.9±0.1	650	690	731	1.63±0.1	3.88±0.05	5.51±0.05
EH-IDTBR	10.3±0.1	650	673	675	1.68±0.1	3.90±0.05	5.58±0.05

*EA*, electron affinity; *IP*, ionization potential.

Measurements were carried out in:

^*^CHCl_3_ solution.

^†^Thin film spin-coated from 10 mg ml^−1^ chlorobenzene solution.

^‡^Thin film annealed at 130 °C for 10 min.

^§^Cyclic voltammetry carried out on the as-cast thin film with 0.1 M TBAPF_6_ electrolyte in acetonitrile.

^||^Estimated from the electrochemical *EA* and the optical *E*_g_.

**Table 2:** Photovoltaic performance of optimized EH-IDTBR:P3HT and O-IDTBR:P3HT solar cells.

	***J***_**sc**_ **(mA cm**^−2^**)**	***V***_**oc**_ **(V)**	**FF**	**PCE (%)**
O-IDTBR:P3HT	13.9±0.2	0.72±0.01	0.60±0.03	6.30±0.1
EH-IDTBR:P3HT	12.1±0.1	0.76±0.01	0.62±0.02	6.00±0.05

FF, fill factor; PCE, power conversion efficiency. Devices were measured under simulated AM1.5G illumination at 100 mW cm^−2^ with average values obtained from 8 to 10 devices.
