# Supplementary material for: High-efficiency and air-stable P3HT-based polymer solar cells with a new non-fullerene acceptor
Source: Nat Commun. 2016 Jun 9;7:11585. doi: 10.1038/ncomms11585 (PMC4906164; doi:10.1038/ncomms11585)
Supplement: Supplementary Information — Supplementary Figures 1-15 and Supplementary Tables 1-4 [file ncomms11585-s1.pdf]

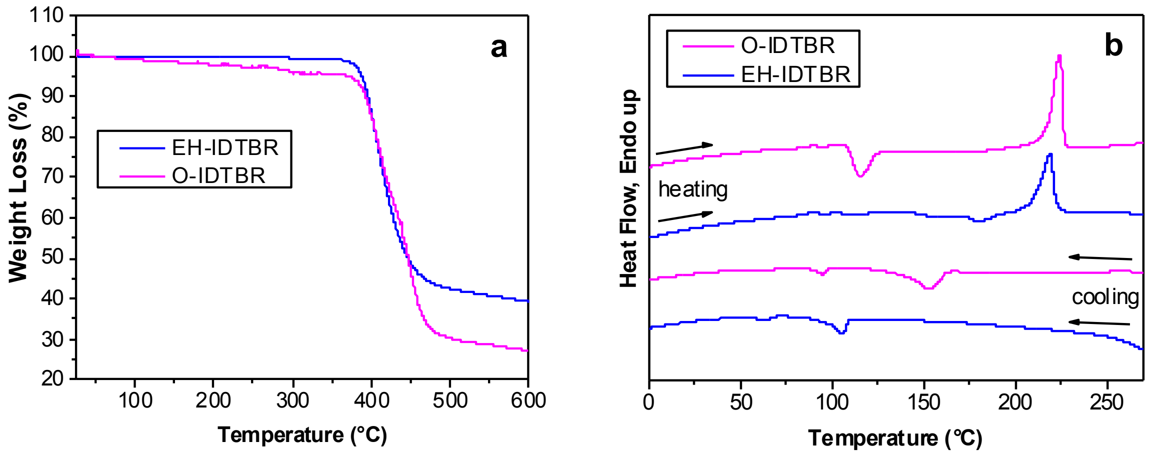

Supplementary Figure 1. (a) Thermogravimetric analysis (10 °C min^{-1}) and (b) differential scanning calorimetry (5 °C min^{-1}) of EH-IDTBR and O-IDTBR measured under nitrogen. Thermograms are offset vertically for clarity.

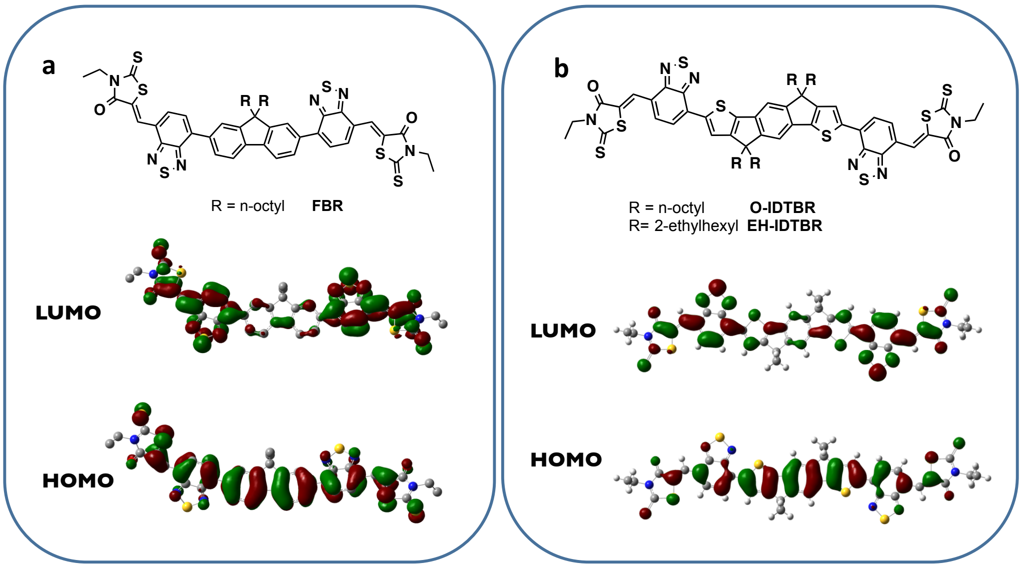

Supplementary Figure 2. Chemical structures and minimum energy conformations of (a) FBR and (b) IDTBR calculated (with methyl replacing n-octyl or 2-ethylhexyl groups) using Gaussian (B3LYP/6-31G*) to visualize the LUMO and HOMO distributions.

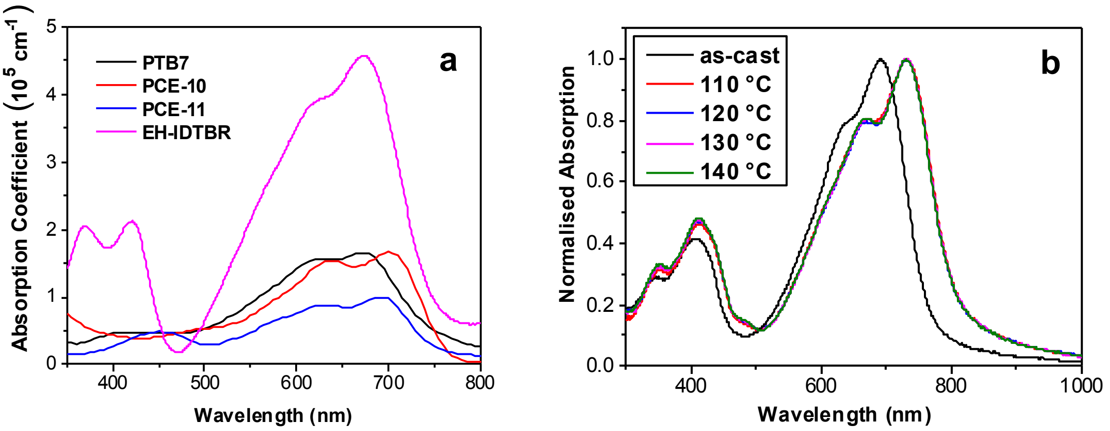

Supplementary Figure 3. (a) Absorption coefficients *α* of EH-IDTBR in the thin film compared with a selection of low bandgap donor polymers (structures shown in Supplementary Figure 16), where *α* was calculated with the equation $\alpha = 1/d*\ln(1/T)$; (b) UV-vis absorption spectra of O-IDTBR thin films spin-coated from 10 mg ml^{-1} chlorobenzene solution, as-cast and with 10 min annealing at different temperatures.

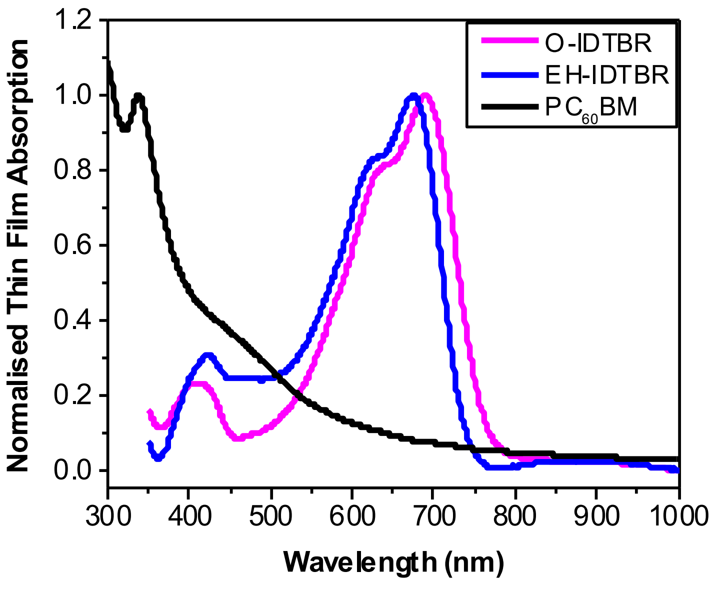

Supplementary Figure 4: Normalised UV-vis absorption spectra of as-cast IDTBR thin films compared with that of $PC_{60}BM$.

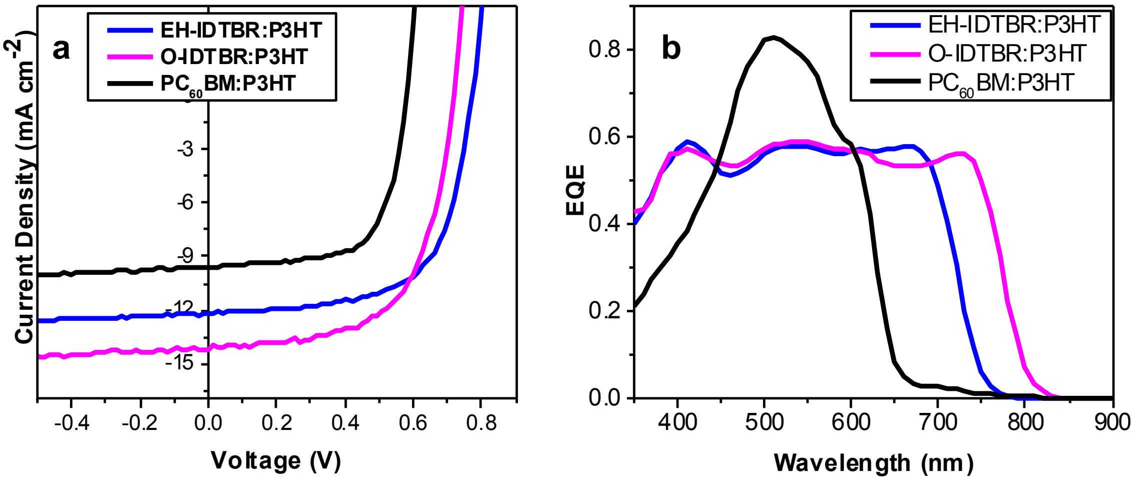

Supplementary Figure 5: (a) *J-V* characteristics and (b) EQE spectra for IDTBR:P3HT devices compared to reference $PC_{60}BM$:P3HT device measured at 100 mW cm^{-2} illumination.

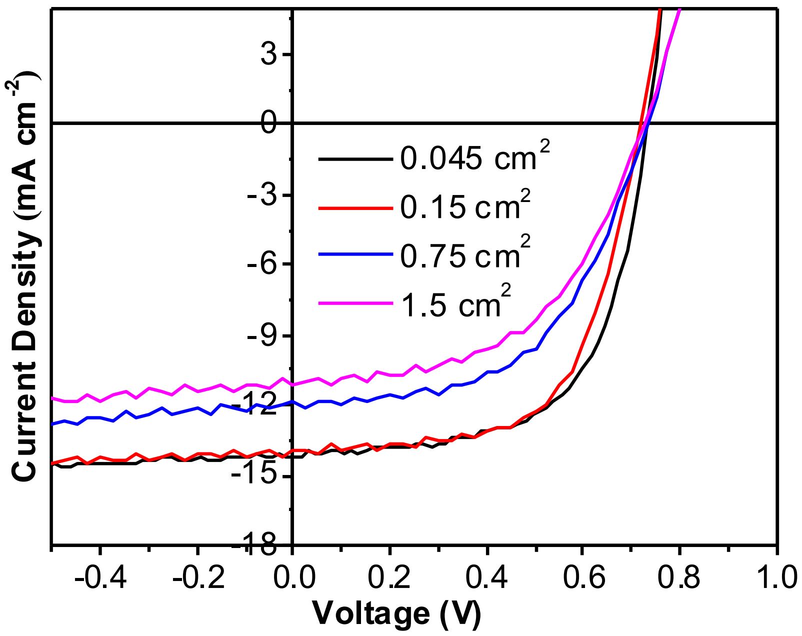

Supplementary Figure 6. *J-V* characteristics of O-IDTBR:P3HT devices with different active areas measured under 100 mW cm^{-2} illumination.

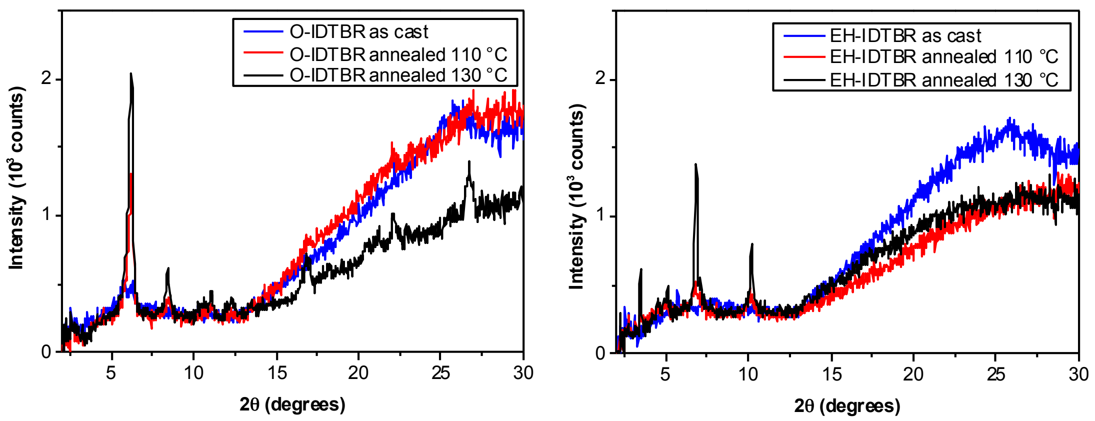

Supplementary Figure 7. Specular XRD of (a) O-IDTBR and (b) EH-IDTBR films as-cast and with annealing at 110 °C and 130 °C.

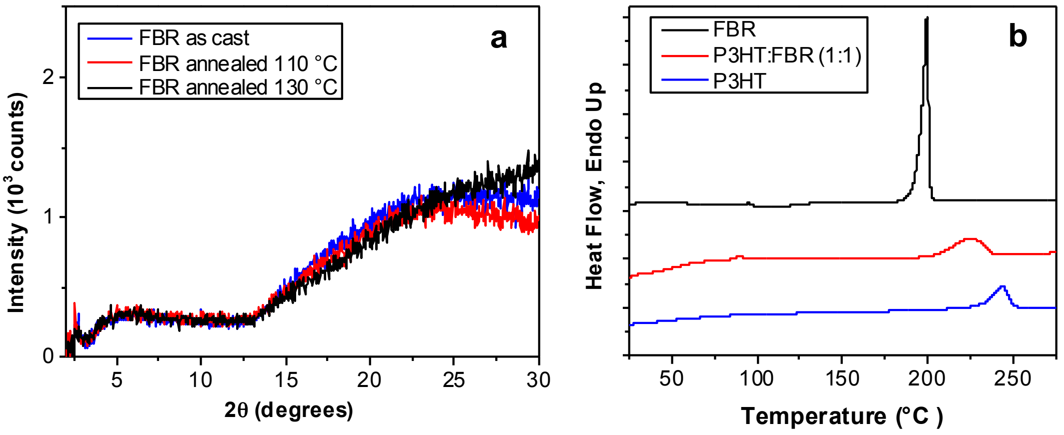

Supplementary Figure 8. (a) Specular XRD of FBR films as-cast and with annealing at 110 °C and 130 °C; (b) DSC first heating cycles measured at 5 °C min^{-1} on drop-cast samples of FBR, P3HT and P3HT:FBR (1:1). Thermograms are offset vertically for clarity.

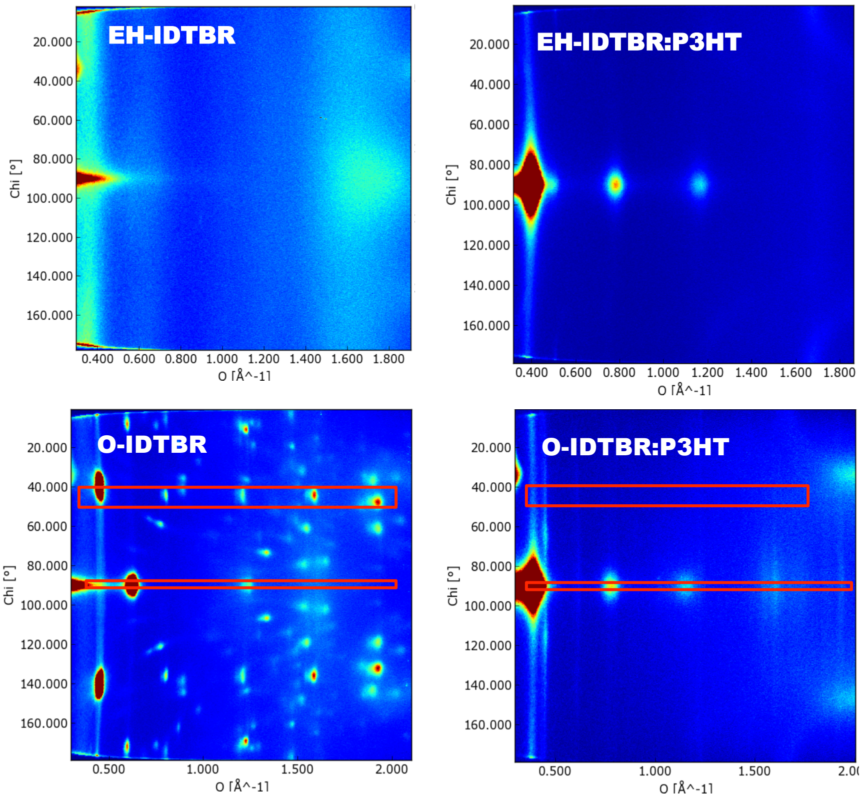

Supplementary Figure 9. Chi-Q plots of (top) EH-IDTBR and EH-IDTBR:P3HT blend (bottom) O-IDTBR and O-IDTBR:P3HT blend, for which the diffraction intensity is integrated in two ranges of Chi = 87.5-92.5° and 40-50° and plotted against the whole wave vector Q.

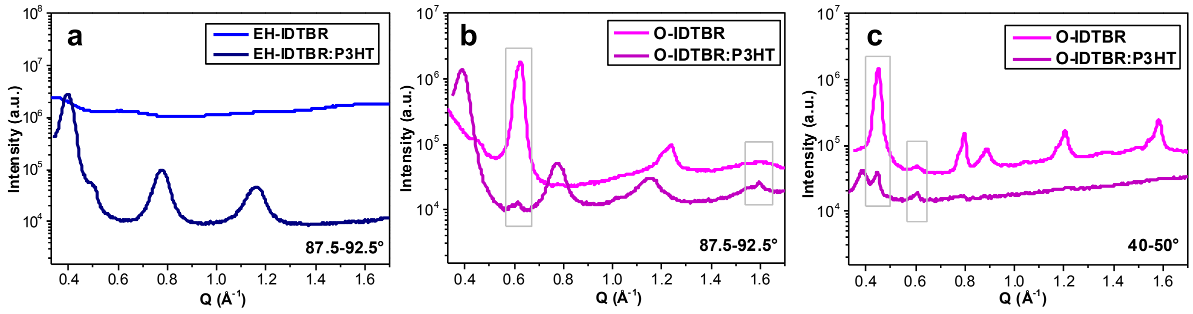

Supplementary Figure 10. Line cuts from GIXRD chi-Q plots of (a) EH-IDTBR and EH-IDTBR:P3HT blend at 87.5-92.5°; (b) O-IDTBR and O-IDTBR:P3HT blend at 87.5-92.5°; (c) O-IDTBR and O-IDTBR:P3HT blend at 40-50°.

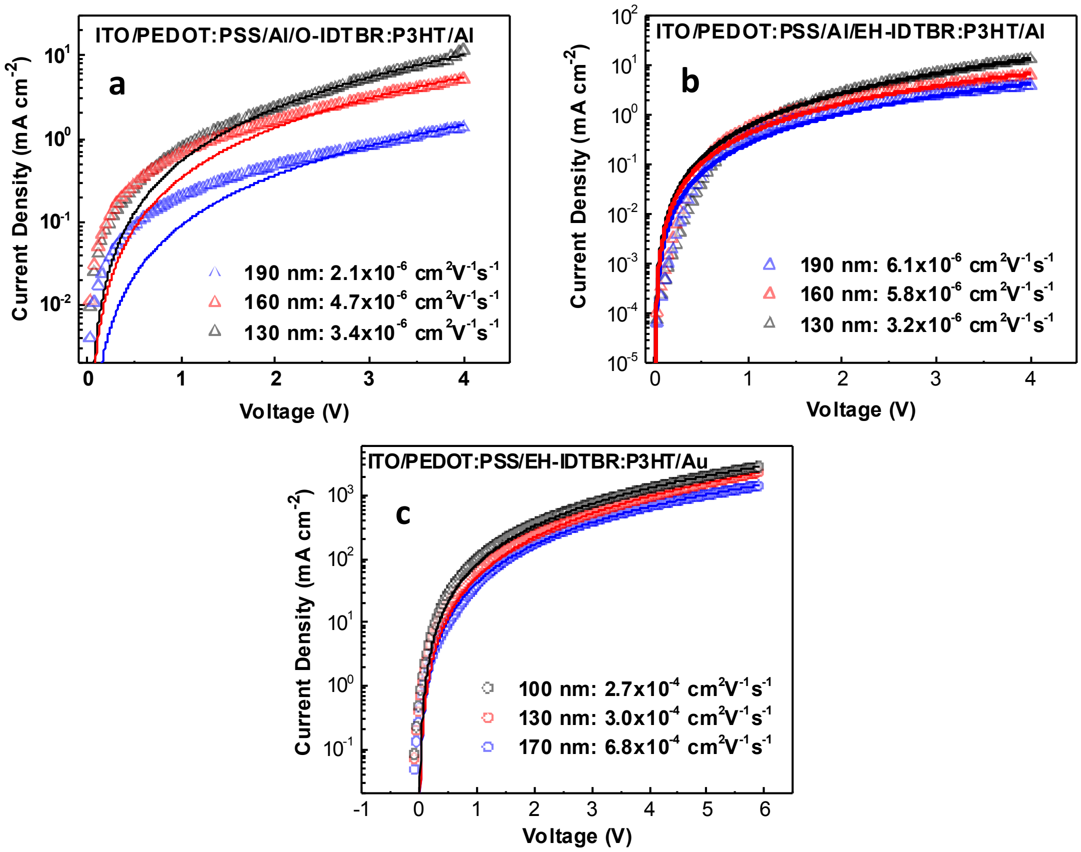

Supplementary Figure 11. Current-voltage characteristics of electron-only devices of different thickness in log-lin representation for (a) O-IDTBR:P3HT and (b) EH-IDTBR:P3HT blends and of hole-only devices of different thickness for (c) EH-IDTBR:P3HT blends. The solid lines represent fits to the experimental data (open triangles in (a) and (b) and open dots in (c)) according to the Mott-Gurney law assuming space charge-limited currents in these devices. Note that for EH-IDTBR:P3HT the hole mobility is about two orders higher than the electron mobility.

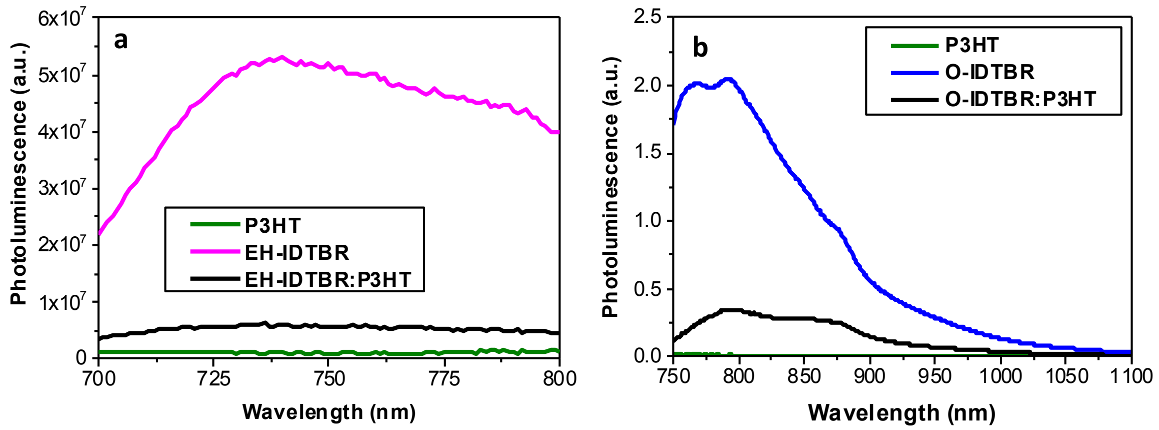

Supplementary Figure 12: Photoluminescence spectra of EH-IDTBR, O-IDTBR and annealed EH-IDTBR:P3HT (130 °C for 10 min) and O-IDTBR:P3HT (130 °C for 20 min) blends excited at 680 nm. Note that P3HT does not absorb at this excitation wavelength. All the spectra are corrected for film absorption.

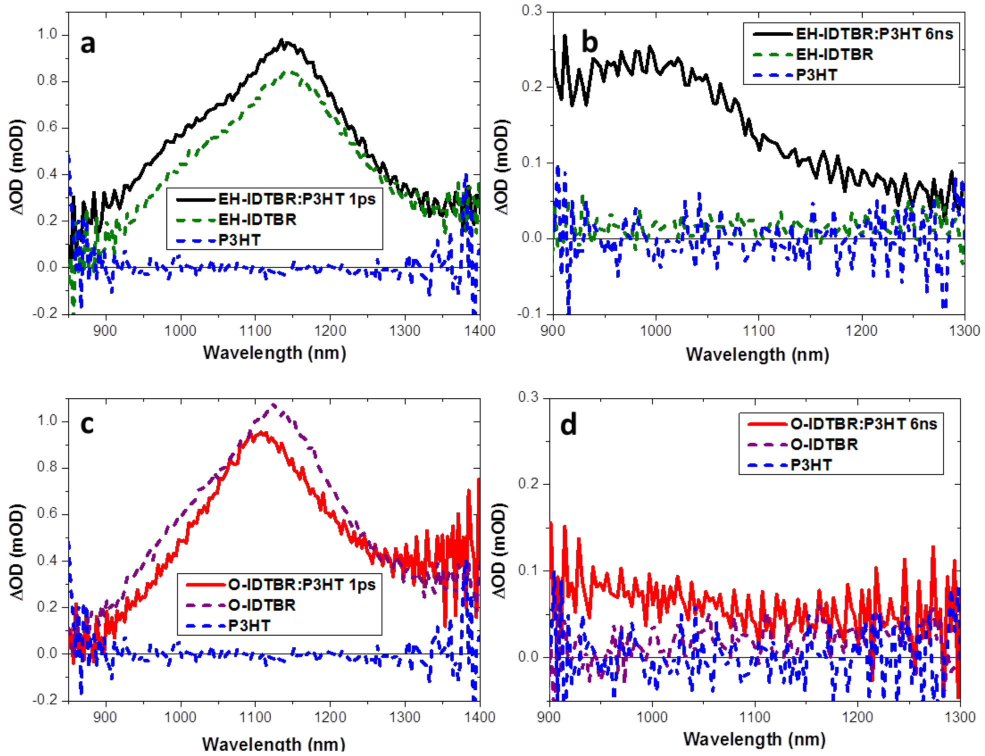

Supplementary Figure 13. Femtosecond-transient absorption spectra of (a), (b) EH-IDTBR:P3HT and (c), (d) O-IDTBR:P3HT blends and pristine counterparts excited at 680 nm with 2μJ cm^{-2} density in N_2 atmosphere at (a), (c) 1 pico-second (exciton signature) and (b), (d) 6 nano-second (polaron signature).

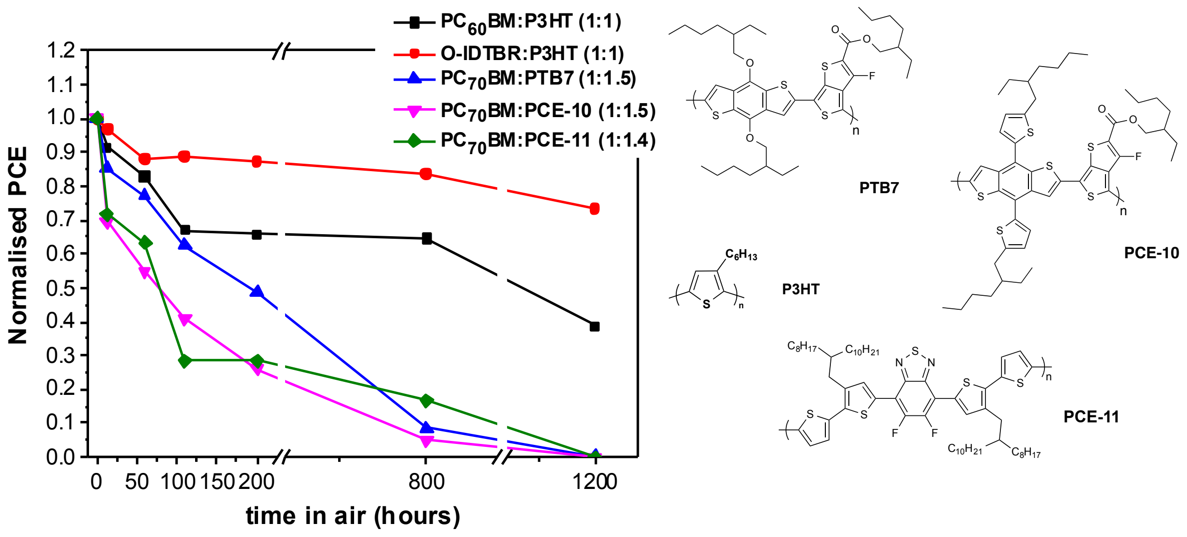

Supplementary Figure 14. Oxidative stability of **O-IDTBR**:P3HT devices (normalised PCE values) compared with other high performace polymer:fullerene systems (polymer structures shown). Devices were exposed to air over the course of 1200 hr.

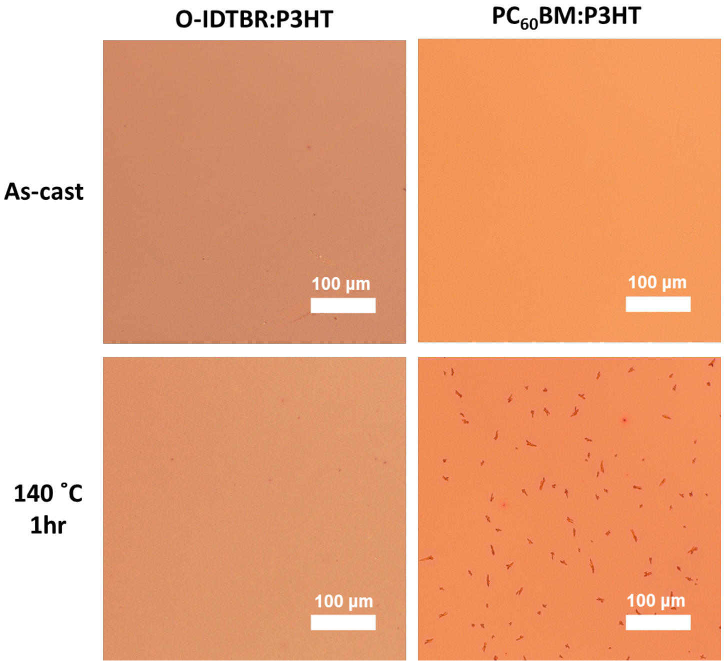

Supplementary Figure 15. Optical microscopy of O-IDTBR:P3HT blends in comparison with $PC_{60}BM$:P3HT blends. Films were prepared on ITO/ZnO coated glass substrates according to procedures for the device active layers, and then annealed for 1 h under inert atmosphere.

Supplementary Table 1. Optoelectronic properties of $PC_{60}BM$ as measured in this study.

ε [10^4 M^{-1} cm^{-1}]$^{a)}$	λ_{max} film [nm] $^{b)}$	E_g opt. [eV]$^{b)}$	EA [eV] $^{c)}$	IP [eV] $^{d)}$
0.39 (400 nm)	333	2.05	4.10	6.15

Measurements were carried out in $^{a)}$ $CHCl_3$ solution; $^{b)}$ thin film spin-coated from 10 mg ml^{-1} chlorobenzene solution; $^{c)}$ cyclic voltammetry carried out on the as-cast thin film with 0.1 M $TBAPF_6$ electrolyte in acetonitrile; $^{d)}$ estimated from the EA and the optical E_g.

Supplementary Table 2. Photovoltaic characteristics of $PC_{60}BM$:P3HT reference devices

	J_{sc} [mA cm^{-2}]	V_{oc} [V]	FF	PCE [%]
**$PC_{60}BM$:P3HT**	9.59	0.58	0.67	3.73

Supplementary Table 3. Photovoltaic performance of O-IDTBR:P3HT OPV devices tested for differerent active areas under 100 mW cm^{-2} illumination.

Area of Device	J_{sc} [mA/cm^2]	V_{oc} [V]	FF	PCE [%]
0.045 cm^2	14.1	0.73	0.62	6.4
0.15 cm^2	13.9	0.72	0.63	6.3
0.75 cm^2	11.9	0.73	0.55	4.8
1.5 cm^2	11.1	0.73	0.53	4.3

Supplementary Table 4: Device parameters for the cells used in photo-CELIV measurements measured under 100 mW cm^{-2} illumination.

	J_{sc} [mA cm^{-2}]	V_{oc} [V]	FF	PCE [%]
**O-IDTBR:P3HT**	13.5	0.73	0.60	5.91
**EH-IDTBR:P3HT**	12.0	0.76	0.61	5.56
